# A Novel Potent Carrier for Unconventional Protein Export in *Ustilago maydis*

**DOI:** 10.3389/fcell.2021.816335

**Published:** 2022-01-10

**Authors:** Magnus Philipp, Kai P. Hussnaetter, Michèle Reindl, Kira Müntjes, Michael Feldbrügge, Kerstin Schipper

**Affiliations:** Institute for Microbiology, Heinrich Heine University Düsseldorf, Düsseldorf, Germany

**Keywords:** luciferase, anti-Sars-CoV2 nanobody, unconventional secretion, *Ustilago maydis*, sybody

## Abstract

Recombinant proteins are ubiquitously applied in fields like research, pharma, diagnostics or the chemical industry. To provide the full range of useful proteins, novel expression hosts need to be established for proteins that are not sufficiently produced by the standard platform organisms. Unconventional secretion in the fungal model *Ustilago maydis* is an attractive novel option for export of heterologous proteins without *N*-glycosylation using chitinase Cts1 as a carrier. Recently, a novel factor essential for unconventional Cts1 secretion termed Jps1 was identified. Here, we show that Jps1 is unconventionally secreted using a fusion to bacterial β-glucuronidase as an established reporter. Interestingly, the experiment also demonstrates that the protein functions as an alternative carrier for heterologous proteins, showing about 2-fold higher reporter activity than the Cts1 fusion in the supernatant. In addition, Jps1-mediated secretion even allowed for efficient export of functional firefly luciferase as a novel secretion target which could not be achieved with Cts1. As an application for a relevant pharmaceutical target, export of functional bi-specific synthetic nanobodies directed against the SARS-CoV2 spike protein was demonstrated. The establishment of an alternative efficient carrier thus constitutes an excellent expansion of the existing secretion platform.

## Introduction

The market for recombinant proteins like biopharmaceuticals is steadily increasing (Walsh 2018). As one example, the number of monoclonal antibody therapeutics entering phase 3 clinical trials has risen from 39 in 2014 to 88 in 2020 (Reichert 2015; Kaplon and Reichert 2021). Protein secretion into the culture broth is an excellent strategy for the production of recombinant proteins because it supports straight-forward and inexpensive downstream processing (Nicaud et al., 1986; Flaschel and Friehs 1993). In eukaryotes, proteins are mostly targeted via the endomembrane system by N-terminal signal peptides for secretion (Viotti 2016). By contrast, the term unconventional secretion describes protein export that does not occur via the classical endomembrane system including endoplasmic reticulum and Golgi apparatus (Nickel 2010). Various routes for such alternative secretion events exist, including direct transfer across the plasma membrane via transporters or self-sustained translocation or vesicular pathways where membrane vesicles are hitchhiked for export (Nickel 2010; Rabouille 2017).

Unconventional export of chitinase Cts1 in yeast cells of the fungal model *Ustilago maydis* is coupled to cytokinesis in a lock-type mechanism (Reindl et al., 2019). Upon formation of the daughter cell at one growth pole of the cigar shaped mother cell, Cts1 is targeted to the so-called fragmentation zone delimited at the mother-daughter neck by consecutive formation of two septa (Langner et al., 2015). Here, the chitinase participates in separation of the two cells likely by degrading the remnant cell wall (Langner et al., 2015). Two septation factors, guanine nucleotide exchange factor (GEF) Don1 and kinase Don3, are essential for formation of the secondary septum and for Cts1 secretion (Weinzierl et al., 2002; Aschenbroich et al., 2019). Furthermore, a recently identified potential anchoring factor, Jps1, is crucial for chitinase localization and export (Reindl et al., 2020).

Importantly, unconventional Cts1 secretion can be exploited for co-export of heterologous proteins (Stock et al., 2012). Circumventing the classical secretion system is advantageous for the production of distinct proteins, because it avoids post-translational modifications like *N*-glycosylation occurring in the endomembrane system. In addition, there is no apparent size limitation (Stock et al., 2012). Successful examples are secretion of functional enzymes like β-glucuronidase or β-galactosidase, and antibody formats like single-chain variable fragments (scFv) or nanobodies (Stock et al., 2012; Sarkari et al., 2014; Terfrüchte et al., 2017; Reindl et al., 2020). While the secretion system is operational for several target proteins, low yields in the µg per liter range are currently limiting its applicability (Terfrüchte et al., 2017). Recently, major improvements were achieved by the generation of protease-deficient production strains, usage of strong constitutive promoters and medium optimization (Sarkari et al., 2014; Terfrüchte et al., 2018). However, novel strategies to further advance the system are needed.

In the present study we demonstrate that Jps1 is a novel potent carrier for co-export of heterologous proteins. We observed improved overall yields of secreted protein and export of firefly luciferase that was not functionally secreted via Cts1-fusions. As a proof-of-principle for pharmaceutical proteins we exported functional nanobodies directed against the receptor-binding domain (RBD) of the SARS-CoV2 spike protein. The novel carrier thus constitutes an important improvement of our expression system towards a competitive production platform.

## Results

### Jps1 is a Potent New Carrier for Unconventional Protein Export

**FIGURE 1:**
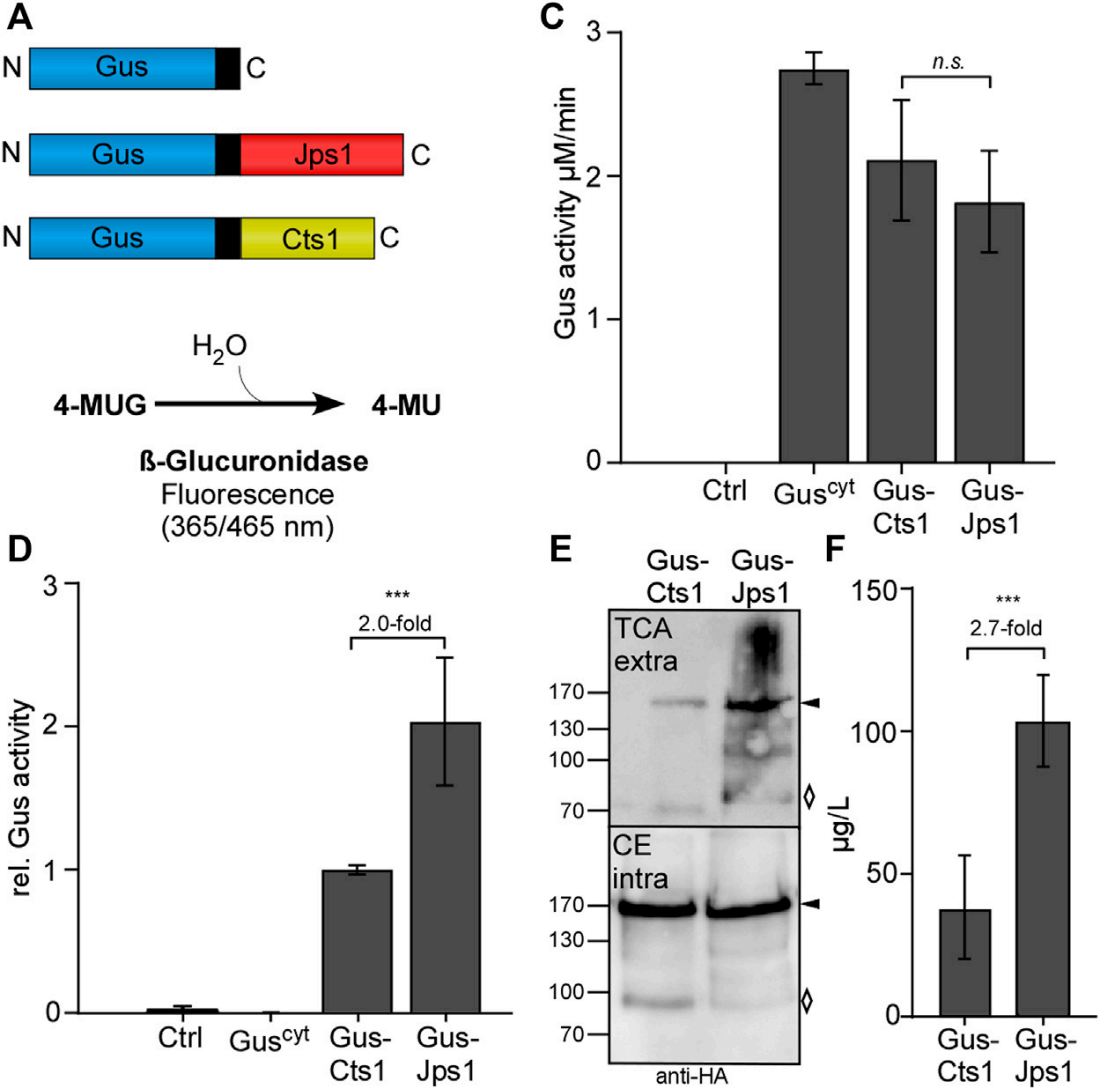
Jps1 is unconventionally secreted and serves as an alternative carrier for Gus export. **(A)** Schematic display of the proteins expressed to study unconventional secretion. Cytoplasmic Gus (Gus^cyt^) is used as a lysis control (top). Gus-Jps1 (middle) and Gus-Cts1 (bottom) harbor the respective carrier proteins at the C-terminus. All proteins carry an SHH (double Strep, ten times His, triple HA) tag indicated in black (Sarkari et al., 2014). All schemes are drawn to scale. **(B)** Enzymatic reaction mediated by β-glucuronidase. 4-methyl-umbeliferyl-β-D-glucuronide (4-MUG) and H_2_O are converted to 4-methyl-umbelliferone which is a fluorescent molecule (365 nm excitation/465 nm emission). **(C)** Determination of intracellular Gus activity. Progenitor strain AB33P8Δ (Ctrl) and AB33 Gus^cyt^ expressing cytoplasmic Gus were included as controls. The experiment was conducted in three biological replicates. **(D)** Comparative extracellular Gus activity of strains using either Cts1 or Jps1 as a carrier. Enzyme activities were normalized to average values of the strain secreting Gus-Cts1. AB33P8Δ and AB33 Gus^cyt^ were used as a negative and lysis controls, respectively. The experiment was conducted in three biological replicates. **(E)** Representative Western blot analysis of Gus-Cts1 and Gus-Jps1 secretion. Extracellular protein was enriched from culture supernatants by TCA precipitation. Intracellular protein levels were visualized by cell extracts. Western blots show 1 ml of precipitated supernatants (TCA) and 10 μg cell extract (CE). Full length protein signal indicated by arrows, degradation bands with a rhombus. **(F)** Quantification of secreted protein using Western blot analysis. Supernatants of strains producing Gus-Jps1 or Gus-Cts1 were enriched by TCA precipitation and subjected to Western blot analysis. Signal intensities were compared to defined protein amounts of Multiple Tag protein (GenScript Piscataway, NJ, United States) included in the same gel. Bars show extrapolated protein amounts in µg/L. Western blots used for the analysis, see Supplementary Figure S3. Three biological replicates are shown; error bars in figures **(C)**, **(D)**, and **(F)** indicate standard deviation. Definition of statistical significance (***): *p*-value < 0.05. *p*-value derived from Student’s unpaired *t*-test.

Previous experiments had shown that Jps1 co-localizes with Cts1 in the fragmentation zone (Reindl et al., 2020), suggesting that it might also be unconventionally secreted. To study this, we applied the well-established β-glucuronidase (Gus) reporter system (Figure 1A,B). This bacterial enzyme is largely inactivated upon secretion through the eukaryotic endomembrane system. By contrast, it is released in a functional state via unconventional secretion in yeast cells of *U. maydis* (Stock et al., 2012). To assay unconventional secretion of Jps1, a strain expressing a Gus-Jps1 fusion protein was generated in the background of the octuple protease-deletion laboratory strain AB33P8Δ (Figure 1A) (Terfrüchte et al., 2018). Microscopic analysis revealed that yeast cells expressing Gus-Jps1 did not show any morphological differences as compared to the progenitor (Supplementary Figures S1, S2). The Gus-Jps1 fusion did also not disturb Cts1 function as detected by determining extracellular chitinase activity of AB33P8Δ/Gus-Jps1 which was similar to the activity detected in a strain expressing Gus-Cts1 (Supplementary Figure S1). Subsequently, intra- and extracellular Gus activity was determined (Figures 1C,D). The progenitor strain AB33P8Δ was used as a negative control, while a strain expressing intracellular Gus served as a lysis control (AB33 Gus^cyt^) (Stock et al., 2012). High Gus activity was present in cell extracts of all strains harboring the Gus enzyme but not in the progenitor AB33P8Δ lacking the enzyme (Figure 1C). Importantly, Gus activity was also detected in the supernatant of Gus-Jps1 expressing strains but not for the lysis control, confirming unconventional secretion of Jps1 (Figure 1D). At the same time, this experiment demonstrates, that Jps1—similar to Cts1—is able to act as a carrier for heterologous proteins. Notably, extracellular Gus activity levels were increased by about 2-fold in culture supernatants of Gus-Jps1 compared to Gus-Cts1 expressing strains (Figure 1D), suggesting that Jps1 might constitute a more effective carrier than Cts1. Both strains were also compared in terms of growth speed and strain fitness using online monitoring in a BioLector device (m2p-labs, Baesweiler, Germany) (Funke et al., 2010). The progenitor strain AB33P8Δ as well as AB33P8∆/Gus-Cts1 and AB33P8∆/Gus-Jps1 showed similar proliferation patterns and doubling times of about 3 h during the exponential growth phase when incubated in CM medium supplemented with 1% glucose (Supplementary Figure S2). Thus, Jps1 constitutes a promising candidate for a novel potent carrier for heterologous proteins.

To assay secretion on the protein level, Western blot analyses were conducted. These experiments showed that extracellular amounts of Gus-Jps1 were markedly increased as compared to Gus-Cts1, while intracellular levels were comparable. This confirms that Jps1 is secreted with enhanced efficiency in relation to Cts1 (Figure 1E, Supplementary Figure S3). To quantify this result distinct amounts of Multiple Tag protein (GenScript Biotech, Piscataway, NJ, United States) were included (Supplementary Figure S4). Quantification of the Western blot signals revealed that Gus-Cts1 levels in the supernatant reach concentrations of 38 μg/L while Gus-Jps1 is present at about 103 μg/L (about 2.7-fold increase; Figure 1F). In summary, these results demonstrate that Jps1 can deal as a powerful carrier for heterologous proteins with elevated levels in comparison to Cts1.

### *don3* Induced Secretion Further Enhances Gus-Jps1 Secretion

**FIGURE 2:**
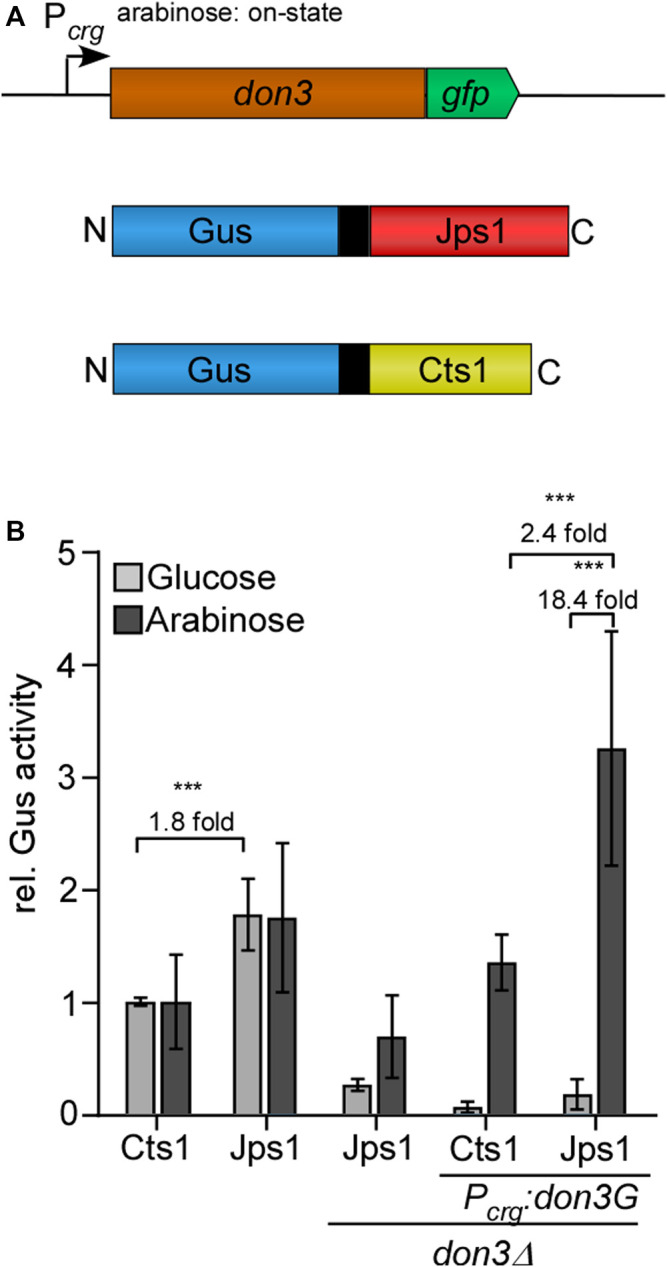
Inducible secretion of Gus-Jps1 via transcriptional regulation of *don3*. **(A)** Schematic display of the inducible secretion system. *don3-gfp* is expressed under control of the arabinose-inducible promoter P_*crg*._ Under glucose conditions the promoter is in its “off state”, unconventionally secreted proteins under control of P_*oma*_ are thus expressed but not secreted. Under arabinose condition the promoter is in its “on state” and proteins are secreted. Gus is fused to either Cts1 or Jps1 including an internal SHH tag (double Strep, ten times His, triple HA). **(B)** Gus activity in culture supernatants of AB33 derivatives expressing Gus-Cts1 or Gus-Jps1 and their *∆don3* variants. Enzymatic activity was normalized to average values of positive controls secreting Gus-Cts1 constitutively. The diagram represents the results of three biological replicates. Error bars depict standard deviation. Fold change of induced cultures depicted over brackets. Definition of statistical significance (***): *p*-value < 0.05. *p*-value derived from Student’s unpaired *t*-test.

Recently, we have established a system that allows for the induction of unconventional secretion via regulation of kinase Don3, a gatekeeper of the fragmentation zone (Hussnaetter et al., 2021). To this end we used a arabinose-inducible promoter to control *don3* expression, which is prerequisite for secondary septum formation (Weinzierl et al., 2002). Unconventional secretion is only functional with a functional fragmentation zone consisting of two septa (Aschenbroich et al., 2019). Here we reproduced these findings using Jps1 as a carrier as demonstrated by a strain which carried genetic modifications for transcriptional induction of *don3* and expressed the Gus-Jps1 reporter as read-out (Figure 2A,B) (Hussnaetter et al., 2021). Although we observed a slightly higher background activity in arabinose cultures, the induction was more than 18-fold and thus, significantly higher than for using Cts1 as a carrier protein, showing about 12-fold induction (Figure 2B). Furthermore, Gus-activity was elevated 2.4-fold compared to induced Gus-Cts1 secretion and more than 3-fold compared to regular Gus-Cts1 secretion. Hence, inducible Jps1 constitutes a powerful tool for unconventional secretion of heterologous proteins. Jps1 enables export of functional firefly luciferase.

### Jps1 Enables Export of Functional Firefly Luciferase

**FIGURE 3:**
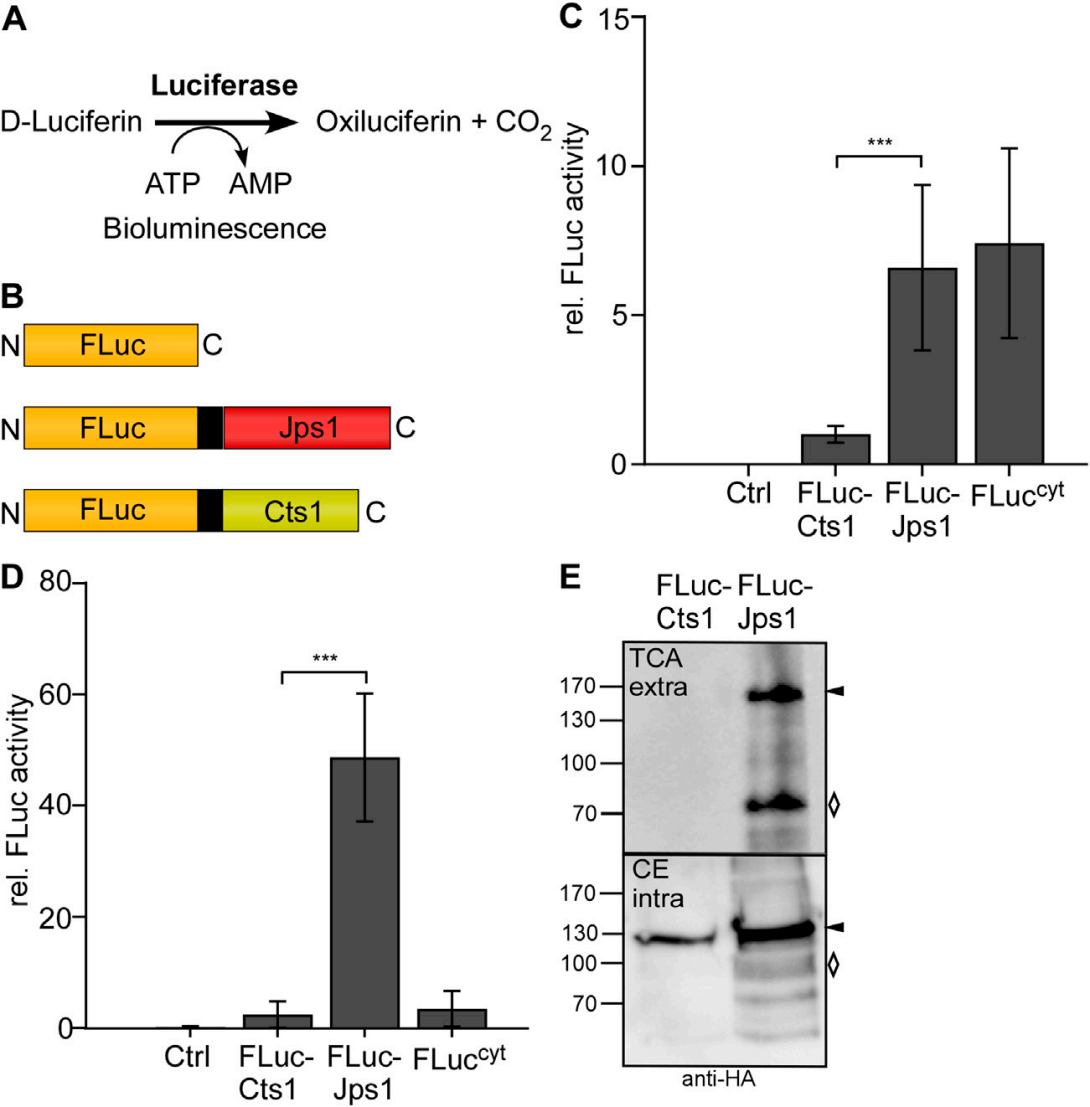
Efficient Jps1-mediated export of firefly luciferase as a new reporter for unconventional secretion. **(A)** Schematic display of the proteins expressed to study unconventional secretion. Cytoplasmic FLuc (FLuc^cyt^) was used as a lysis control (top). FLuc-Jps1 (middle) and FLuc-Cts1 (bottom) harbor the respective carrier proteins at the C-terminus. All proteins carry an SHH tag indicated in black (Sarkari et al., 2014). All schemes are drawn to scale. **(B)** Enzymatic reaction mediated by firefly luciferase: D-Luciferin and ATP are converted to oxiluciferin, AMP and CO_2_. During this reaction excited intermediates emit energy in the form of light that can be detected as bioluminescence. **(C)** Comparison of intracellular FLuc activity of the strains AB33P8Δ/FLuc-Cts1 and AB33P8Δ/FLuc-Jps1. Enzymatic activity was normalized to average values of strain secreting FLuc-Cts1. The progenitor strain AB33P8Δ was used as a negative control. Strain AB33 FLuc^cyt^ with intracellular FLuc expression dealt as positive control. Three biological replicates are shown. **(D)** Comparison of extracellular FLuc activity of strains harboring either Cts1 or Jps1 as a carrier. Enzymatic activity was normalized to average values of strain secreting FLuc-Cts1. Strain AB33 FLuc^cyt^ with intracellular FLuc expression dealt as lysis control. Three biological replicates are shown. Error bars in figures **(C)** and **(D)** indicate standard deviation. Definition of statistical significance (***): *p*-value < 0.05. *p*-value was derived from Student’s unpaired *t*-test. **(E)** Representative Western blot of FLuc-Cts1 and FLuc-Jps1. Secreted protein was enriched from the supernatant by TCA precipitation. Intracellular protein levels were visualized by cell extracts. Western blots show 1 ml of precipitated supernatants (TCA) and 10 μg cell extracts (CE). Full length protein signals indicated by arrows, degradation bands with a rhombus.

*Photinus pyralis* luciferase FLuc was recently established for intracellular use in *U. maydis* (Müntjes et al., 2020). Bioluminescence would be a straight-forward alternative read-out for unconventional secretion because the signal can be detected directly from the culture broth while the established reporters Gus and β-galactosidase (LacZ) require more elaborate biochemical assays. Further advantages are low background signals and the use of the inexpensive substrate D-luciferin Figure 3A (Miska and Geiger 1987). To test bioluminescence as a read-out for unconventional secretion, an expression strain producing FLuc-Cts1 was generated in the background of the octuple protease deletion strain (AB33P8∆/FLuc-Cts1). Similarly, a FLuc-Jps1 expressing strain was generated (AB33P8∆/FLuc-Jps1) to evaluate the effect of the alternative carrier (Figure 3B). AB33 producing intracellular luciferase (FLuc^Cyt^) was used as a positive control in all assays (Müntjes et al., 2020). Monitoring of proliferation revealed that growth speed was slightly reduced in AB33P8∆/FLuc-Jps1 with a doubling time of 3.5 h, compared to the progenitor strain AB33P8Δ and AB33P8∆/FLuc-Cts1 showing doubling times of 3 h in the exponential growth phase (Supplementary Figure S2). The slight reduction might eventually be caused by a minor increase in the number of abnormal cells growing in clusters in the FLuc-Jps1 expressing strain (Supplementary Figure 2C). Luciferase assays showed that intracellular activity was very low in the FLuc-Cts1 expressing strain compared to the strain producing cytoplasmic FLuc, while levels of Fluc-Jps1 expressing strains were comparable to the cytoplasmic control showing significant activity (Figure 3C). Importantly, in culture supernatants the observed effect was even more pronounced and extracellular FLuc activity for the strain producing FLuc-Jps1 was about 48-fold higher than activity of FLuc-Cts1 secreting cells for which no significant difference to the control strain could be observed (Figure 3D). These results were confirmed in Western blot analyses. While intracellular levels of FLuc-Cts1 were reduced in comparison to FLuc-Jps1 which showed an about 1.8-fold higher signal intensity, only FLuc-Jps1 was detectable in supernatants (Figure 3E; Supplementary Figure S3). This demonstrates that not only expression of FLuc-Cts1 was impaired but also detectable Cts1 based secretion was absent. The reason for the differential performance of the Cts1 and Jps1 fusions with FLuc remains unclear. The size of the FLuc-Cts1 fusion protein is likely not affecting its unconventional secretion, since larger fusions had been successfully exported in earlier studies (Stock et al., 2012). Eventually, structural interferences or other unknown features of this particular fusion lead to reduced protein production or its instability. These results further emphasize the advantage of having a second carrier for the unconventional secretion system at hands.

### Unconventional Secretion of Functional Antibodies Against Sars-CoV2-Receptor Binding Domain

**FIGURE 4:**
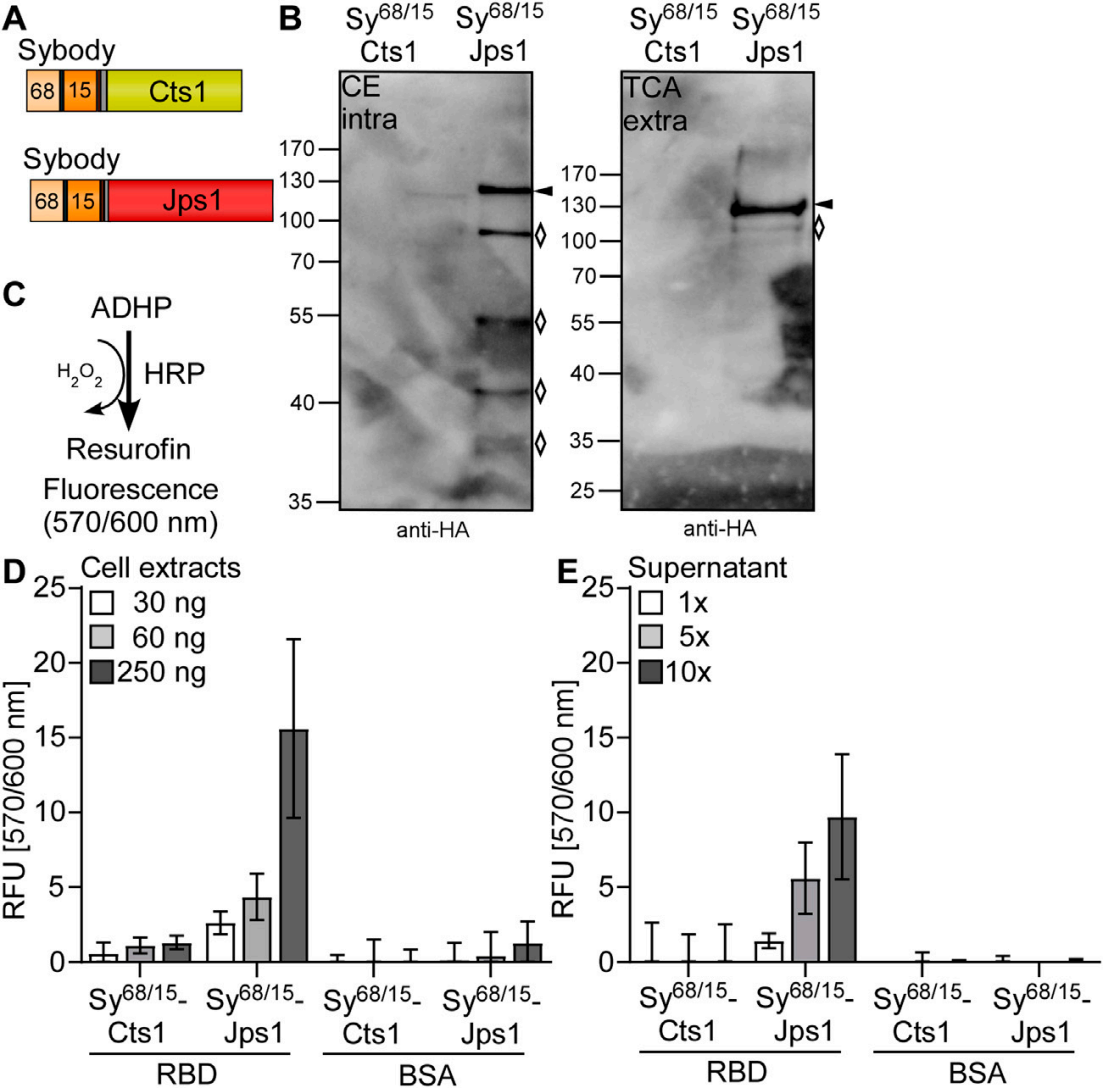
Export of functional bi-specific Sars-CoV2 sybodies using Jps1 as a carrier for unconventional secretion. **(A)** Bi-specific anti SARS-CoV2 spike-RBD sybodies sy#15 and sy#68 (Walter et al., 2020) were tagged with a 10x His tag and fused to either Cts1 (top) or Jps1 (bottom) via a TEV protease cleavage site and an HA-tag. **(B)** Detection reaction for ELISA: Colorless 10-acetyl-3,7-dihydrophenoxazine (ADHP) is converted by horseradish peroxidase (HRP) using H_2_O_2_ to resurofin, a purple substance that emits strong fluorescence (excitation 570 nm, emission 600 nm). **(C)** Representative Western blot analyses of Sy^68/15^-Cts1 and Sy^68/15^-Jps1. Secreted protein was enriched from the supernatant by TCA precipitation. Intracellular protein levels were visualized by cell extracts. Western blots show 1 ml of precipitated supernatants (TCA) and 10 μg cell extracts (CE). Full length protein signals indicated by arrows, degradation bands with rhombi. **(D)** ELISA of cell extracts: 1 µg of RBD was immobilized per well. 1 µg BSA dealt as a negative control. Baseline was established by a well coated with RBD and only treated with anti-HA and anti-mouse-HRP. Serial dilutions of *U. maydis* cell extracts (30 ng, 60 ng, 250 ng per well) were applied in technical triplicates both to RBD and BSA coated wells. Detection was carried out with the before mentioned anti-HA-mouse and anti-mouse-HRP antibodies. Three biological replicates are shown. Error bars indicate standard deviation of biological replicates. **(E)** ELISA of protein purified from supernatants: ELISA wells were coated, and reactions detected as described in **(D)**. Culture supernatants containing sybody-fusion proteins were subjected to Nickel^2+^-NTA IMAC and subsequently concentrated up to 10-fold. Serial dilutions of supernatants (1-fold, 5-fold, 10-fold concentrated supernatant) were mixed with blocking solution and added to ELISA wells in technical triplicates. Three biological replicates are shown. Error bars indicate standard deviation for biological replicates.

Next, we tested unconventional secretion of nanobodies directed against the SARS-CoV2 spike protein receptor binding domain (RBD) as a timely example of pharmaceutically relevant targets. Therefore, strains were generated in which two synergistic synthetic nanobodies (sybodies) directed against the Sars-CoV2 spike-RBD were combined (Walter et al., 2020). The bi-specific sybody was tagged with a 10× His-linker for purification and fused to either Cts1 or Jps1 for unconventional secretion (AB33P8Δ/Sy^68/15^-Cts1 and AB33P8Δ/Sy^68/15^-Jps1) (Figure 4A). Western blot analyses confirmed that both fusion proteins were synthesized. However, Sy^68/15^-Cts1 was produced at a lower level compared to Sy^68/15^-Jps1. The latter showed stronger degradation than observed for other Jps1 fusion proteins (see above). In supernatants only a very faint signal was present for Sy^68/15^-Cts1 while for Sy^68/15^-Jps1 a stronger signal and less degradation than in cell extracts was detected (Figure 4B). Quantification revealed an increase of about 18-fold in signal intensity for the Jps1 full-length fusion compared to the Cts1 full-length fusion (Supplementary Figure S3). Subsequently, the antigen-binding activity of the sybody was determined via direct confrontation with spike-RBD immobilized on ELISA plates and subsequent detection with an antibody sandwich Figure 4C. Immobilized bovine serum albumin (BSA) dealt as a negative control. ELISA experiments using cell extracts demonstrated that both sybody-fusion proteins were functional in detecting the cognate antigen. While the activity of Sy^68/15^-Cts1 was only slightly above baseline, Sy^68/15^-Jps1 showed strong volumetric activity (Figure 4D). Next, sybody-fusion proteins were IMAC purified from culture supernatants and applied to ELISA in up to 10-fold concentrated solutions Figure 4E. While no activity could be observed for Sy^68/15^-Cts1, Sy^68/15^-Jps1 showed volumetric binding activity on the antigen, confirming the secretion of the functional sybody fusion protein. Thus, pharmaceutically relevant nanobodies were exported in their functional form using Jps1 as a carrier for unconventional secretion.

## Discussion

Here we successfully evaluated the potential anchoring factor Jps1 as a novel carrier for the export of heterologous proteins by unconventional secretion in *U. maydis*. Carrier proteins are ubiquitously used in fungal protein expression systems based on conventional secretion (Fleissner and Dersch 2010). This is mainly due to the observation that homologous proteins like hydrolytic enzymes are secreted with very high titers compared to heterologous targets (Nevalainen and Peterson 2014). In our system, similar to the previously used carrier chitinase Cts1, Jps1 was fused to the C-terminus of heterologous target proteins to mediate their export via the fragmentation zone. Of note, one exception identified during this study was the reporter enzyme LacZ: Here, a LacZ-Cts1 fusion is functional and unconventionally secreted (Reindl et al., 2020) while strains producing the respective LacZ-Jps1 fusion showed growth retardation and were lacking detectable LacZ activity and LacZ-Jps1 protein in the culture supernatant (results not shown). We anticipate that this could be related to the formation of tetramers by LacZ which interfere with Jps1 but not with Cts1 secretion; however, this hypothesis needs to be verified. Nevertheless, the discovery of a second carrier for unconventional secretion in *U. maydis* is a very favorable addition to our expression system (Reindl et al., 2019; Wierckx et al., 2021): The choice between the two fusion proteins, Cts1 and Jps1, will greatly enhance the repertoire of our secretion targets. Jps1 proofed valuable for the export of proteins that were not secreted at significant levels as Cts1 fusions and showed promising secretion levels for these targets. This is for example true for the firefly luciferase FLuc or the bi-specific sybodies that were only secreted efficiently when fused to Jps1. As a positive side effect, the FLuc-Jps1 fusion protein is a valuable alternative that allows a quick and inexpensive quantification of unconventional secretion via Jps1 in future studies (Wider and Picard 2017; Branchini et al., 2018). On the contrary, the intrinsic feature of chitin binding of Cts1 is very attractive as a tool which can be developed for efficient *in situ* protein purification from culture broth (Terfrüchte et al., 2017). Hence, both carriers show distinct advantages that can be exploited depending on the actual demands.

In line with our results, different carriers show varying efficiencies in other fungal systems. For example, glycoamylase or α-amylase have been described as a powerful tool for heterologous protein secretion in filamentous fungi like Aspergilli (Ward et al., 1990; Nakajima et al., 2006). Similarly, the choice of the conventional signal peptide for efficient entry into the endoplasmic reticulum has been described as a key factor for improving conventional secretion (Xu et al., 2018; Wang et al., 2020). While existence of a signal peptide remains elusive for lock-type unconventional secretion (Stock et al., 2012), it is conceivable that other unconventionally secreted proteins are still to be discovered that might constitute even more powerful carriers. Currently, we do not have a precise idea on why Jps1 mediates export of heterologous proteins more effectively than Cts1. Further studies on the molecular roles of Jps1 during Cts1 secretion might resolve this question in the future. Notably, unconventional secretion was also observed for septation factor Don3 (Aschenbroich et al., 2019) which may thus serve as such alternative carrier. However, Gus activity levels of unconventionally secreted Gus-Don3 are minute compared to Gus-Cts1, suggesting that it does not constitute a promising alternative (Aschenbroich et al., 2019). Hence, it is important to further study the mechanism of lock-type secretion and in particular, to identify further players that localize to the fragmentation zone for export during cytokinesis (Reindl et al., 2019; Wierckx et al., 2021).

The successful synthesis and functional export of nanobodies directed against the RBD of the surface spike protein of the SARS-CoV2 virus is a timely new addition to the repertoire of secreted targets. The current pandemics situation underpinned that it is important to develop novel methodology for quick, specific, and sensitive detection and treatment of viral infections in the future. On the one hand nanobodies are potent proteins for antigen detection (Muyldermans 2013) and thus very promising tools in the context of SARS-CoV2 detection. On the other hand, antibody-based pharmaceuticals like Casirivimab and Imdevimab are already used to treat COVID-19 infection (Sun and Ho 2020). Therefore, besides application in virus diagnostics, nanobodies directed against SARS-CoV2 could potentially even become novel pharmaceutical targets for therapeutic approaches (Dubey et al., 2020). The unique system of unconventional secretion in *U. maydis* now offers new possibilities for nanobody production without the risk of undesired modifications by *N*-glycosylation (Stock et al., 2012). This would eliminate the necessity to humanize llama derived nanobodies for safe use as pharmaceuticals to avoid allergic reaction in patients (Vincke et al., 2009; Dong et al., 2020). To achieve this, both the unconventional secretion system and specifically the production and application of nanobodies via this system have to be optimized, for example by further multimerization to increase valency and affinity (Wichgers Schreur et al., 2020; Koenig et al., 2021). By the establishment of a new carrier and export of functional SARS-CoV2 nanobodies we have thus laid a solid foundation for further exploitation and application of lock-type unconventional secretion.

## Material and Methods

### Molecular Biology Methods

All plasmids (pUMa/pUx vectors) generated in this study were obtained using standard molecular biology methods established for *U. maydis* including Golden Gate and Gibson cloning (Brachmann et al., 2004; Gibson 2011; Gibson et al., 2009; Terfrüchte et al., 2014). All plasmids were verified by restriction analysis and sequencing. Oligonucleotides applied for cloning are listed in Table 1. Genomic DNA of *U. maydis* strain UM521 was used as template for PCR reactions. The genomic sequence for this strain is stored at the EnsemblFungi database (EnsemblFungi). The generation of plasmids pUMa3329_Δupp1_P_crg_-eGfp-T_nos_-natR, pUMa2113_pRabX1-P_oma__gus-SHH-cts1, pUMa2240_Ip_Poma-his-αGfpllama-ha-Cts1-CbxR and pUMa3771_Δupp3_Potef_FLuc_NatR has been described previously (resulting strains, see Table 2). For the generation of pUMa3012_Ip_Poma_Gus-SHH-Jps1_CbxR the *jps1 gene* (*umag_03776*) was amplified from genomic DNA using primers oMB372 and oMB373 with AscI and ApaI hydrolyzation sites. Subsequently, the backbone of pUMa2113_Ip_Poma_Gus-SHH-Cts1_CbxR was used for restriction ligation cloning and *jps1* was inserted into the backbone instead of *cts1*. pUMa4131_Ip_Poma_FLuc-SHH-Cts1_CbxR was generated by amplification of the *U. maydis* dicodon-optimized *P. pyralis fluc* gene from pUMa3771_Δupp3_Potef_FLuc_NatR using oAB297 and oAB298 with BamHI and SfiI hydrolyzation sites. pUMa2113_Ip_Poma_Gus-SHH-Cts1_CbxR was then hydrolyzed with BamHI and SfiI and *fluc* was inserted into the backbone instead of *gus* via restriction/ligation cloning. A restriction/ligation cloning approach was applied for pUMa4566_Ip_Poma_FLuc-SHH-Jps1_CbxR. *jps1* was excised from pUMa3012_Ip_Poma_Gus-SHH-Jps1_CbxR using AscI and ApaI and inserted into pUMa4131_Ip_Poma_FLuc-SHH-Cts1_CbxR, also hydrolyzed with AscI and ApaI. pUx1_Ip_Poma-Sy#68-his-Sy#15-ha-Cts1-CbxR was generated by amplification of genes *sy* ^*#68*^ and *sy* ^*#15*^ (Walter et al., 2020) from a synthetic gBlock (Integrated DNA Technology, Coralville, Iowa, United States) using primers oAB908 and oAB909 for *sy* ^*#15*^ adding BamHI and SpeI hydrolyzation sites and oCD234 and oCD235 for *sy* ^*#68*^ with complementary overhangs for Gibson cloning. Subsequently, pUMa2240_Ip_Poma-his-αGfpllama-ha-Cts1-CbxR (Terfrüchte et al., 2017) was hydrolyzed with BamHI and SpeI and gene *sy* ^*#15*^ was inserted via restriction ligation cloning, replacing *αgfpllama* and thereby generating pUMa4678. pUMa4678 was then hydrolyzed with BamHI and the sequence encoding for sy^#68^ was inserted via Gibson cloning (Gibson et al., 2009), generating pUx1. For the generation of pUx8 *jps1* was excised from pUMa3012 using AscI and ApaI and inserted into the AscI and ApaI hydrolyzed backbone of pUx1.

**TABLE 1:** DNA oligonucleotides used in this study.

Designation	Nucleotide sequence (5′- 3′)
oMB372_jps1_fw	TTA​GGC​GCG​CCA​TGC​CAG​GCA​TCT​CC
oMB373_jps1_rev	TTA​GGG​CCC​TTA​GGA​TTC​CGC​ATC​GAT​TGG​GG
oMF502_ip_fw	ACG​ACG​TTG​TAA​AAC​GAC​GGC​CAG
oMF503_ip_rev	TTC​ACA​CAG​GAA​ACA​GCT​ATG​ACC
oAB297_fluc_fw	AAA​TTG​GAT​CCA​TGG​AGG​ACG​CCA​AGA​ACA​TCA​AG
oAB298_fluc_rev	AAT​AGG​CCG​CGT​TGG​CCA​CGG​CGA​TCT​TGC​CAC​CCT​T
oAB908_sy^#15^_fw	ATA​TAG​GAT​CCA​TGG​CGG​CCC​ATC​ACC​ACC​ATC​ACC​ACC​ATC​ACC​ACC​ATC​ATA​TGC​AGG​TGC​AGC​TCG
oAB909_sy^#15^_rev	ATA​TAA​CTA​GTC​GAG​ACG​GTG​ACC​TGG​GTG​C
oCD234_sy^#68^_fw	CTA​CCT​TAC​TCT​ATC​AGG​ATC​ATG​CAG​GTG​CAG​CTC​GTC​G
oCD235_sy^#68^_rev	GGT​GAT​GGG​CCG​CCA​TGG​ATC​CCG​AGA​CGG​TGA​CCT​GGG​TGC

**TABLE 2:** *U. maydis* strains used in this study.

Strains	Relevant genotype/Resistance	Strain collection no. (UMa^a^)	Plasmids transformed/Resistance^b^	Manipulated locus (*umag* gene identifier)	Progenitor (UMa^a^)	References
AB33	*a2 P*_*nar*_ *bW2bE1*	133	pAB33	*b*	FB2 (55)	Brachmann et al. (2004)
	*PhleoR*					
AB33 Gus-Cts1	*a2 P*_*nar*_ *bW2bE1 PhleoR*	1289	pUMa2113/CbxR	*ip*	133	Sarkari et al. (2014)
	*ip* ^*S*^ *(P*_*oma*_ *gus:shh:cts1)ip* ^*R*^ *CbxR*					
AB33don3Δ/Gus-Cts1	*a2 P*_*nar*_ *bW2bE1 PhleoR*	1742	pUMa2717/HygR	*umag_05543 (don3)*	1289	Aschenbroich et al. (2019)
	*ip* ^*S*^ *(P*_*oma*_ *gus:shh:cts1)ip* ^*R*^ *CbxR*					
	*umag_don3Δ_HygR*					
AB33don3Δ	*a2 P*_*nar*_ *bW2bE1 PhleoR*	2028	pUMa2717/HygR	*umag_05543 (don3)*	133	Aschenbroich et al. (2019)
	*umag_don3Δ_HygR*					
AB33don3Δ/P_crg_don3-gfp/Gus-Cts1	*a2 P*_*nar*_ *bW2bE1 PhleoR*	2302	pUMa3330/NatR	*umag_02178* (*upp1*)	1742	Aschenbroich et al. (2019)
	*ip* ^*S*^ *(P*_*oma*_ *gus:shh:cts1)ip* ^*R*^ *CbxR*					
	*umag_don3Δ_HygR*					
	*upp1:(P*_*crg*_ *don3:gfp) NatR*					
AB33P8∆Gus-Cts1	*a2 P*_*nar*_ *bW2bE1 PhleoR*	2418	pUMa2113	*Ip*	2413	Terfrüchte et al. (2018)
	*FRT10(um04641Δ:hyg)*					
	*FRT11(um03947Δ)*					
	*FRT6(um03975Δ)*					
	*FRT5(um04400Δ)*					
	*FRT3(um11908Δ)*					
	*FRT2(um00064Δ)*					
	*FRTwt[um02178Δ)*					
	*FRT1(um04926Δ) HygR*					
	*ip* ^*S*^ *(P*_*oma*_ *gus:shh:cts1)ip* ^*R*^ *CbxR*					
AB33don3Δ/Gus-Jps1	*a2 P*_*nar*_ *bW2bE1 PhleoR*	2734	pUMa3012	*Ip*	2028	This study
	*ip* ^*S*^ *(P*_*oma*_ *gus:shh:cts1)ip* ^*R*^ *CbxR*					
	*umag_don3Δ_HygR*					
AB33don3Δ/P_crg_don3-gfp/Gus-Jps1	*a2 P*_*nar*_ *bW2bE1 PhleoR*	2776	pUMa3330/NatR	*umag_02178* (*upp1*)	2734	This study
	*ip* ^*S*^ *(P*_*oma*_ *gus:shh:cts1)ip* ^*R*^ *CbxR*					
	*umag_don3Δ_HygR*					
	*upp1:(P*_*crg*_ *don3:gfp) NatR*					
AB33P8∆Gus-Jps1	*a2 P*_*nar*_ *bW2bE1 PhleoR*	2900	pUMa3012	*Ip*	2413	this study
	*FRT10(um04641Δ:hyg)*					
	*FRT11(um03947Δ)*					
	*FRT6(um03975Δ)*					
	*FRT5(um04400Δ)*					
	*FRT3(um11908Δ)*					
	*FRT2(um00064Δ)*					
	*FRTwt[um02178Δ)*					
	*FRT1(um04926Δ) HygR*					
	*ip* ^*S*^ *(P*_*oma*_ *gus:shh:jps1)ip* ^*R*^ *CbxR*					
AB33P8∆ FLuc-Cts1	*a2 P*_*nar*_ *bW2bE1 PhleoR*	3151	pUMa4131	*Ip*	2413	this study
	*FRT10(um04641Δ:hyg)*					
	*FRT11(um03947Δ)*					
	*FRT6(um03975Δ)*					
	*FRT5(um04400Δ)*					
	*FRT3(um11908Δ)*					
	*FRT2(um00064Δ)*					
	*FRTwt[um02178Δ)*					
	*FRT1(um04926Δ) HygR*					
	*ip* ^*S*^ *(P*_*oma*_ *fluc:shh:cts1)ip* ^*R*^ *CbxR*					
AB33P8∆ FLuc-Jps1	*a2 P*_*nar*_ *bW2bE1 PhleoR*	3214	pUMa4566	*ip*		this study
	*FRT10(um04641Δ:hyg)*					
	*FRT11(um03947Δ)*					
	*FRT6(um03975Δ)*					
	*FRT5(um04400Δ)*					
	*FRT3(um11908Δ)*					
	*FRT2(um00064Δ)*					
	*FRTwt[um02178Δ)*					
	*FRT1(um04926Δ) HygR*					
	*ip* ^*S*^ *(P*_*oma*_ *fluc:shh:jps1)ip* ^*R*^ *CbxR*					
AB33P8∆Sy#68/#15-Cts1	*a2 P*_*nar*_ *bW2bE1 PhleoR*	Ux1	pUx1	*ip*	2413	this study
	*FRT10(um04641Δ:hyg)*					
	*FRT11(um03947Δ)*					
	*FRT6(um03975Δ)*					
	*FRT5(um04400Δ)*					
	*FRT3(um11908Δ)*					
	*FRT2(um00064Δ)*					
	*FRTwt[um02178Δ)*					
	*FRT1(um04926Δ) HygR*					
	*ip* ^*S*^ *(P*_*oma*_ *antirbdsybody#68:his:antirbdsybody#15:ha:cts1)ip* ^*R*^ *CbxR*					
AB33P8∆Sy#68/#15-Jps1	*a2 P*_*nar*_ *bW2bE1 PhleoR*	Ux8	pUx8	*ip*	2413	this study
	*FRT10(um04641Δ:hyg)*					
	*FRT11(um03947Δ)*					
	*FRT6(um03975Δ)*					
	*FRT5(um04400Δ)*					
	*FRT3(um11908Δ)*					
	*FRT2(um00064Δ)*					
	*FRTwt[um02178Δ)*					
	*FRT1(um04926Δ) HygR*					
	*ip* ^*S*^ *(P*_*oma*_ *antirbdsybody#68:his:antirbdsybody#15:ha:jps1)ip* ^*R*^ *CbxR*					

a Internal strain collection numbers (UMa/Ux codes).

b Plasmids generated in our working group are integrated in a plasmid collection and termed pUMa, or pUx plus a number as 4-digit number as identifier.

### Strain Generation

*U. maydis* strains used in this study were obtained by homologous recombination yielding genetically stable strains (Bösch et al., 2016) (Table 2). For genomic integrations at the *ip* locus, integrative plasmids were used (Stock et al., 2012). These plasmids contained the *ip* ^r^ allele, promoting carboxin resistance. For integration, plasmids were linearized within the *ip* ^r^ allele to allow for homologous recombination with the *ip* ^*s*^ locus. For transformation, integrative plasmids were hydrolyzed within the *ip* ^r^ locus using the restriction endonuclease SspI, resulting in a linear DNA fragment. For genetic modifications in other loci, plasmids with about 1 kb flanking regions and a resistance cassette were generated (Brachmann et al., 2004; Terfrüchte et al., 2014). For transformation, the insertion cassette was excised from the plasmid backbone using SspI or SwaI (Terfrüchte et al., 2014). For all genetic manipulations, *U. maydis* protoplasts were transformed with linear DNA fragments for homologous recombination. All strains were verified by Southern blot analysis (Southern 1974). For *in locus* modifications the flanking regions were amplified as probes. For *ip* insertions, the probe was obtained by PCR using the primer combination oMF502/oMF503 and the template pUMa260 (Keon et al., 1991; Brachmann et al., 2004). Primer sequences are listed in Table 1.

### Cultivation

U. maydis strains were grown at 28°C in complete medium supplemented with 1% (w/v) glucose (CM-glc) or with 1% (w/v) arabinose (CM-ara) if not described differently (Holliday 1974; Tsukuda et al., 1988). Solid media were supplemented with 2% (w/v) agar agar. Growth phenotypes were evaluated using the BioLector microbioreactor (m2p-labs, Baesweiler, Germany) (Funke et al., 2010). MTP-R48-B(OH) round plates were inoculated with 1.5 ml culture per well and incubated at 1,000 rpm at 28°C. Backscatter light with a gain of 25 or 20 was used to determine biomass.

### Quantification of Unconventional Secretion Using the Gus Reporter

Extracellular Gus activity was determined to quantify unconventional Cts1 secretion using the specific substrate 4-methylumbelliferyl β-D galactopyranoside (MUG, bioWORLD, Dublin, OH, United States)) (Koepke et al., 2011; Stock et al., 2012; Stock et al., 2016). Cell-free culture supernatants were mixed 1:1 with 2× Gus assay buffer (10 mM sodium phosphate buffer pH 7.0, 28 µM β-mercaptoethanol, 0.8 mM EDTA, 0.0042% (v/v) lauroyl-sarcosin, 0.004% (v/v) Triton X-100, 2 mM MUG, 0.2 mg/ml (w/v) BSA) in black 96-well plates. Relative fluorescence units (RFUs) were determined using a plate reader (Tecan, Männedorf, Switzerland) for 100 min at 28°C with measurements every 5 min (excitation/emission wavelengths: 365/465 nm, gain 60). For quantification of conversion of MUG to the fluorescent product 4-methylumbelliferone (MU), a calibration curve was determined using 0, 1, 5, 10, 25, 50, 100, 200 µM MU.

### Determination of Extracellular Cts1 Activity

Extracellular Cts1 activity was analyzed using 4-methylumbelliferyl β-D cellobioside (MUC, Sigma-Aldrich, Billerica, MA, United States) as a substrate (Koepke et al., 2011). Whole cell cultures were mixed 3:7 with KHM Buffer (110 mM CH_3_CO_2_K, 20 mM HEPES, 2 mM MgCl_2_, 2 mM MUC) in black 96 well plates. Relative fluorescence units were determined using a plate reader (Tecan, Männedorf, Switzerland) by fluorescence measurement at 28°C for 100 min every 2 min (360 nm excitation and 450 nm emission, gain 100).

### Quantification of Unconventional Secretion Using Luciferase Reporter

Extra- and intracellular luciferase activity was determined using D-luciferin (Biosynth Carbosynth, Compton, United Kingdom). Cell-free supernatants or whole cell cultures in CM medium were mixed 8:2 with luciferin substrate mix (20 mM tricine, 2.67 mM MgSO_4_×7H_2_O, 0.1 mM EDTA×2 H_2_O, 33.3 mM DTT, 0.524 mM ATP, 0.269 mM acetyl-CoA, 0.467 mM D-luciferin, 5 mM NaOH, 0.264 mM MgCO_3_×5H_2_O) in white 96-well plates. Relative photon count (RPC) was determined using a Mithras LB 940 plate reader (Berthold technologies, Bad Wildbad, Germany) for 20 min with measurements every 20 s.

### Quantification of Unconventional Secretion by Western blot analysis

Gus-Cts1 and Gus-Jps1 secretion was analyzed by trichloroacetic acid (TCA) precipitation of culture broths. 1 ml of cell-free supernatants of cultures grown in Verduyn medium (55.5 mM Glucose, 74.7 mM NH_4_Cl, 0.81 mM MgSO_4_×7H_2_O, 0.036 mM FeSO_4_×7H_2_O, 36.7 mM KH_2_PO_4_, 100 mM MES pH 6.5, 0.051 mM EDTA, 0.025 mM ZnSO_4_×7H_2_O, 0.041 mM CaCl_2_, 0.016 mM H_3_bBO_3_, 6.7 µM MnCl_2_×2H_2_O, 2.3 µM CoCl_2_×6H_2_O, 1.9 µM CuSO_4_×5H_2_O, 1.9 µM Na_2_MoO_4_×2H_2_O, 0.6 µM KI) to an OD_600_ of 3 were chilled on ice and mixed with 400 µl 50% (v/v) TCA solution and incubated on ice at 4°C overnight. Subsequently, precipitated protein pellets were harvested by centrifugation at 11,000 x g at 4°C for 30 min. Supernatants were discarded and pellets were washed with 300 µl of-20°C acetone followed by centrifugation at 11,000 × g at 4°C for 20 min twice. Pellets were dried at room temperature and resuspended in Laemmli buffer containing 0.12 M NaOH. Resuspended pellets were denatured at 95°C for 10 min and then subjected to SDS-PAGE and Western blot analysis. To determine protein concentration obtained by TCA precipitation a standard ladder of 50, 100, 200 and 500 ng of Multiple Tag protein (GenScript Biotech, Piscataway, NJ, United States) was loaded onto the SDS-PAGE next to obtained samples. Western blot signals were quantified using image studio lite version 5.2 (Li-Cor Biosciences, Lincoln, NE, United States) and the standard curve obtained by quantification of Multiple Tag protein signals was used to determine protein concentrations in culture supernatants.

### SDS PAGE and Western Blot Analysis

To verify protein production and secretion in cell extracts and supernatants, respectively, Western Blot analysis was used. 20 ml cultures were grown to an OD_600_ of 1.0 and harvested at 1,500 × g for 5 min in centrifugation tubes. Until further preparation, pellets were stored at −20°C. For preparation of cell extracts, cell pellets were resuspended in 1 ml cell extract lysis buffer (100 mM sodium phosphate buffer pH 8.0, 10 mM Tris/HCl pH 8.0, 8 M urea, 1 mM DTT, 1 mM PMSF, 2.5 mM benzamidine, 1 mM pepstatin A, 2× complete protease inhibitor cocktail (Roche, Sigma/Aldrich, Billerica, MA, United States) and cells were crushed by agitation with glass beads at 2,500 rpm for 12 min at 4°C. After centrifugation (11,000 × g for 30 min at 4°C), the supernatant was separated from cell debris and was transferred to a fresh reaction tube. Protein concentration was determined by Bradford assay (BioRad, Hercules, CA, United States) (Bradford 1976) and 10 µg total protein was used for SDS-PAGE. SDS-PAGE was conducted using 10% (w/v) acrylamide gels. Subsequently, proteins were transferred to methanol activated PVDF membranes using semi-dry Western blotting. SHH-tagged Gus-Cts1 was detected using a primary anti-HA (1:3,000, Millipore/Sigma, Billerica, United States). An anti-mouse IgG-horseradish peroxidase (HRP) conjugate (1:3,000 Promega, Fitchburg, United States) was used as secondary antibody. HRP activity was detected using the Amersham ™ ECL ™ Prime Western Blotting Detection Reagent (GE Healthcare, Chalfont St Giles, United Kingdom) and a LAS4000 chemiluminescence imager (GE Healthcare Life Sciences, Freiburg, Germany).

### IMAC Purification of Supernatants

For the purification of recombinant unconventionally secreted protein from *U. maydis*, cells were grown in CM-glucose (1% w/v) medium buffered with 0.05 M MES pH 6.5.200 ml of culture supernatants were harvested at and OD_600_ of 0.8 by centrifugation at 5,000 × g for 3 min. Harvested supernatants were chilled to 4°C and treated with a protease inhibitor tablet of cOmplete protease inhibitor (Roche, Sigma/Aldrich, Billerica, MA, United States). 2 ml of Nickel^2+^-NTA matrix was equilibrated with 50 ml lysis buffer (10 mM imidazole 50 mM NaH_2_PO_4_, 300 mM NaCl, pH 8.0). 22 ml of 10 times concentrated lysis buffer were added to the supernatants and subsequently Nickel^2+^-NTA matrix was added to the supernatant. The mixture was batched by gentle stirring on a magnetic stirrer at 4°C for 1 h. Following batching supernatant flow-through was discarded via a PD-10 column. Matrix was collected in the PD-10 column during the process. Collected matrix was washed with 50 ml of wash buffer (lysis buffer, 20 mM Imidazole) and protein was eluted with 2 ml elution buffer (lysis buffer, 250 mM imidazole). In the last step supernatants were concentrated via Amicon Ultra 50 k 0.5 ml centrifugal filter devices (Merck Millipore, Burlington, Massachusetts, United States) and the buffer exchanged to PBS (137 mM NaCl, 2.7 mM KCl, 10 mM Na_2_HPO_4_, 1.8 mM KH_2_PO_4_, pH 7.2) and applied for ELISA.

### Enzyme-Linked Immunosorbent Assay

For detection of nanobody binding activity protein adsorbing 384-well microtiter plates (Nunc^®^ Maxisorp™, ThermoFisher Scientific, Waltham, MA, United States) were used. Wells were coated with 1 µg commercially available Sars-CoV2 spike-RBD-domain protein (GenScript Biotech, Piscataway, NJ, United States). 1 µg BSA per well dealt as negative control (NEB, Ipswich, MA, United States). Samples were applied in a final volume of 100 µl coating buffer (100 mM Tris-HCL pH 8, 150 mM NaCl, 1 mM EDTA) per well at 4°C for at least 16 h. Blocking was conducted for at least 4 h at 4°C with 5% (w/v) skimmed milk in coating buffer. Subsequently, 5% (w/v) skimmed milk in PBS was added to defined protein amounts of nanobody samples from cell extracts or purified from culture supernatants and respective controls. 100 µl of sample was added to wells coated with Sars-CoV2 spike-RBD and BSA. The plate was incubated with samples and controls over night at 4°C. After 3x PBS-T (PBS supplemented with 0.05% (v/v) Tween-20, 100 µl per well) washing, a mouse anti-HA antibody (Millipore/Sigma, Billerica, United States) 1: 5,000 diluted in PBS supplemented with skimmed milk (5% w/v) was added (100 µl per well) and incubated for 2 h at room temperature. Then wells were washed again three times with PBS-T (100 µl per well) and incubated with an anti-mouse IgG-horseradish peroxidase (HRP) conjugate (Promega, Fitchburg, United States) (50 µl per well) for 1 h at room temperature [1:5,000 in PBS supplemented with skimmed milk (5% w/v)]. Subsequently wells were washed three times with PBS-T and three times with PBS and incubated with Quanta Red™ enhanced chemifluorescent HRP substrate (50:50:1, 50 µl per well) (ThermoFisher Scientific, Waltham, MA, United States) at room temperature for 15 min. The reaction was stopped with 10 µl per well Quanta RedTM stop solution and fluorescence readout was performed at 570 nm excitation and 600 nm emission using an Infinite M200 plate reader (Tecan, Männedorf, Switzerland).

### Microscopic Analyses

Microscopic analyses were performed with immobilized early-log phase budding cells on agarose patches (3% w/v f. c.) using a wide-field microscope setup from Zeiss (Oberkochen, Germany) Axio Imager M1 equipped with a Spot Pursuit CCD camera (Diagnostic Instruments, Sterling Heights, United States) and the objective lenses Plan Neofluar (×40, NA 1.3), Plan Neofluar (63×, NA 1.25) and Plan Neofluar (100×, NA 1.4). The microscopic system was controlled by the software MetaMorph (Molecular Devices, version 7, Sunnyvale, United States). Image processing including rotating and cropping of images, scaling of brightness, contrast and fluorescence intensities as well as insertion of scale bars was performed with MetaMorph. Arrangement and visualization were performed with Canvas 12 (ACD Systems).

## Data Availability

The original contributions presented in the study are included in the article/Supplementary Material, further inquiries can be directed to the corresponding author.
