# Supplementary material for: A Novel Potent Carrier for Unconventional Protein Export in Ustilago maydis
Source: Front Cell Dev Biol. 2022 Jan 10;9:816335. doi: 10.3389/fcell.2021.816335 (PMC8784666; doi:10.3389/fcell.2021.816335)
Supplement: Supplementary file 1 [file DataSheet6.docx]

Supplementary Material

The supplementary material contains supplementary figures 1-4.

# Supplementary Data


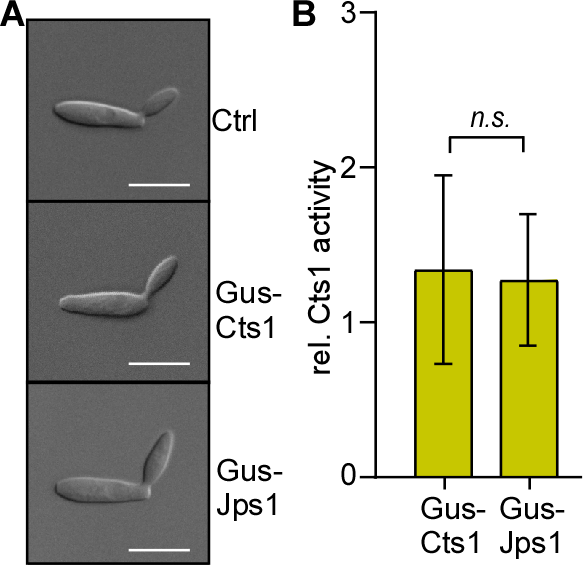


**Supplementary Figure 1. Cell morphology and Cts1 activity of Gus-Cts1 and Gus-Jps1 producing strains. (A)** Microscopic morphology of Gus-Cts1 and Gus-Jps1 expressing strains during yeast-like growth. The progenitor strain AB33P8Δ dealt as reference (Ctrl). Scale bars represent 10 µm. **(B)** Determination of extracellular Cts1 activity in Gus-Jps1 and Gus-Cts1 expressing strains. For determination of extracellular Cts1 activity yeast like growing cultures were incubated with 4-methylumbelliferyl β-D cellobioside. The changes in the relative fluorescence at 360 nm excitation and 450 nm emission was monitored for 90 min. The experiment was conducted in three biological replicates.


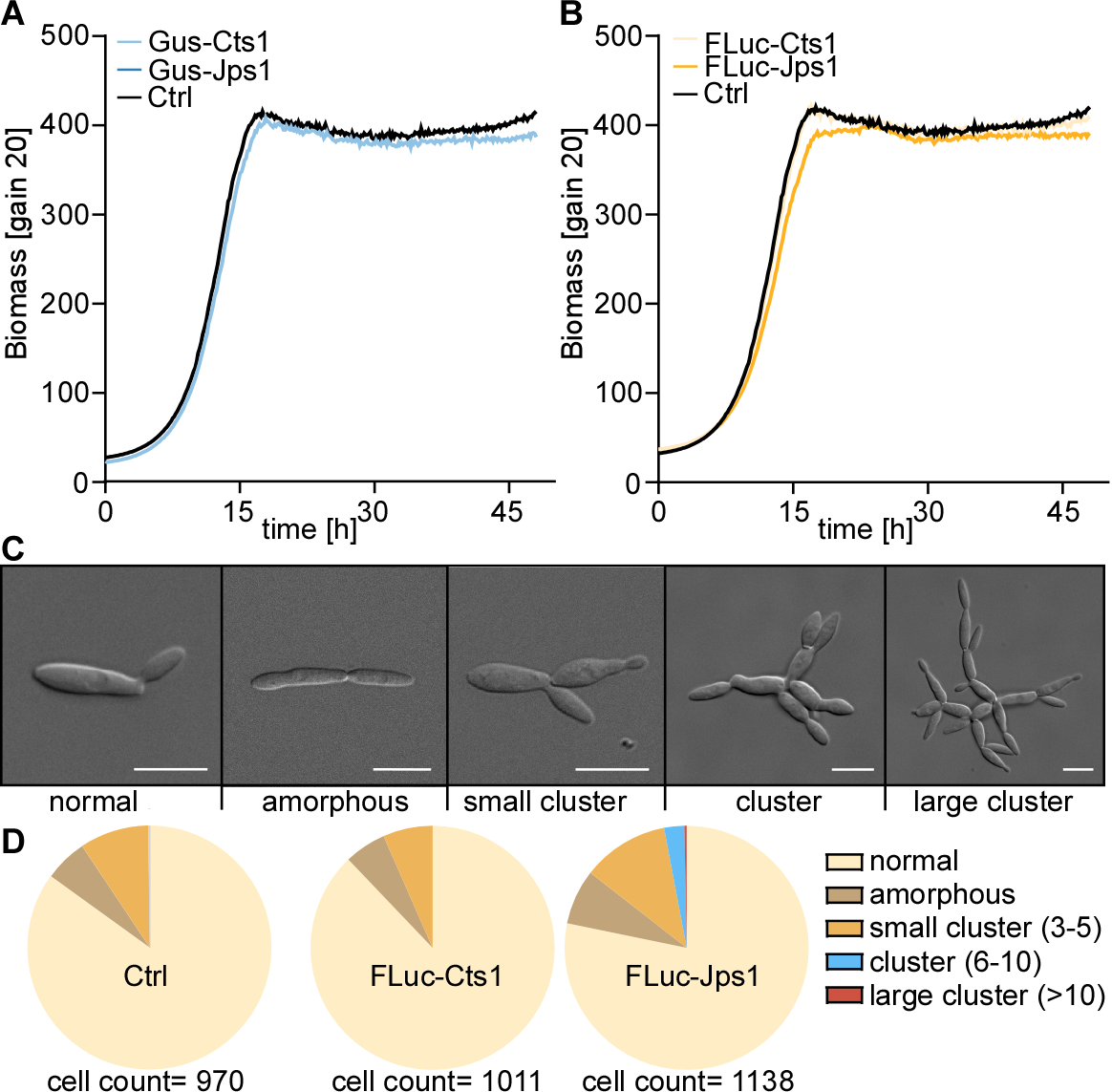


**Supplementary Figure 2. Morphology and fitness of strains exporting firefly luciferase FLuc.** **(A)** Proliferation of strains secreting Gus-Cts1, and Gus-Jps1 was compared to the progenitor strain AB33P8Δ (Ctrl) in a BioLector online monitoring device (m2p-labs, Baesweiler, Germany). The Gus-Cts1 producing strains and the progenitor entered exponential growth phase after a 7 h lag phase and entered stationary phase after 17 h. During exponential growth phase all strains exhibited a doubling time of 3 h. **(B)** Proliferation of strains secreting FLuc-Cts1, and FLuc-Jps1 was compared to the progenitor strain AB33P8Δ (Ctrl) in a BioLector online monitoring device. The FLuc-Jps1 producing strain entered exponential growth phase after 8 h and entered stationary phase after 17 h. Overall biomass was slightly reduced to the progenitor and FLuc-Cts1. Doubling time for Fluc-Jps1 was 3.5 h. **(C)** Representative DIC pictures of different occuring morphologies during yeast-like growth. Left to right: normal cell, amorphous cell, small cluster (3-5 cells), cluster (6-10 cells), large cluster (>10 cells). Scale bars represent 10 µm. **(D)** Morphological quantification of the FLuc-Cts1 and FLuc-Jps1 expressing strains according to morphologies depicted in **(C)**. Progenitor AB33P8Δ dealt as control (Ctrl).


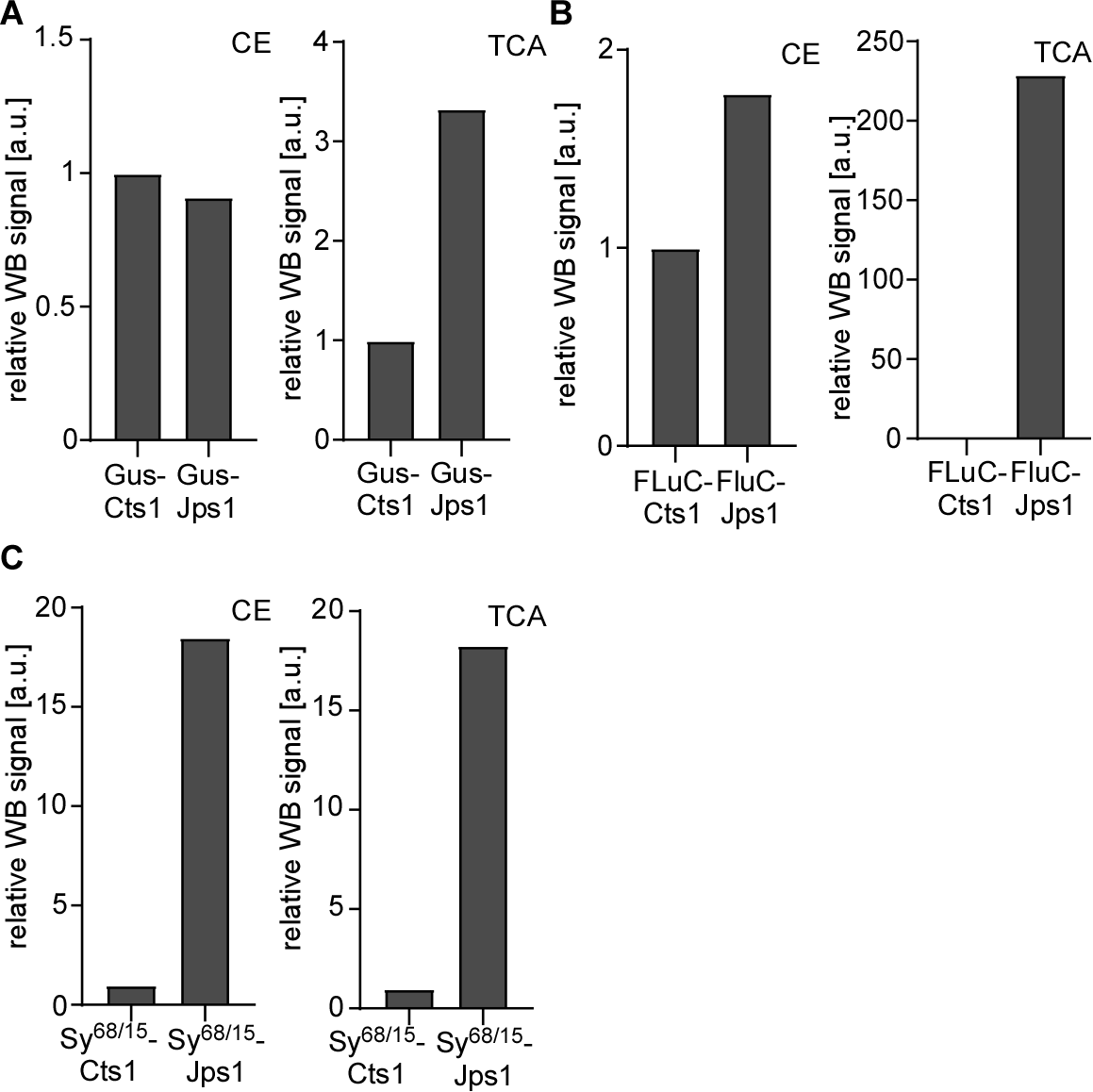


**Supplementary Figure 3. Relative quantification of Western blot signals.** Western blots shown in the main manuscript were analysed with Image Studio Lite ver. 5.2 (Li-Cor Biosciences) to obtain relative signal intensities. Signal intensities were normalised to the intensity of the band of the Cts1 fusion protein in each blot and fold changes in comparison to the other analysed bands are depicted. CE, cell extracts; TCA, TCA-precipitated culture supernatants. **(A)** Relative quantification of Western blots of cell extracts and TCA precipitations of Gus-Cts1 and Gus-Jps1 as shown in Fig. 1 E. **(B)** Relative quantification of Western blots of cell extracts and TCA precipitations of FLuc-Cts1 and FLuc-Jps1 as shown in Fig. 3 E. **(C)** Relative quantification of Western blots of cell extracts and TCA precipitations for Sy^68/15^-Cts1 and Sy^68/15^-Jps1 as shown in Fig. 4C.


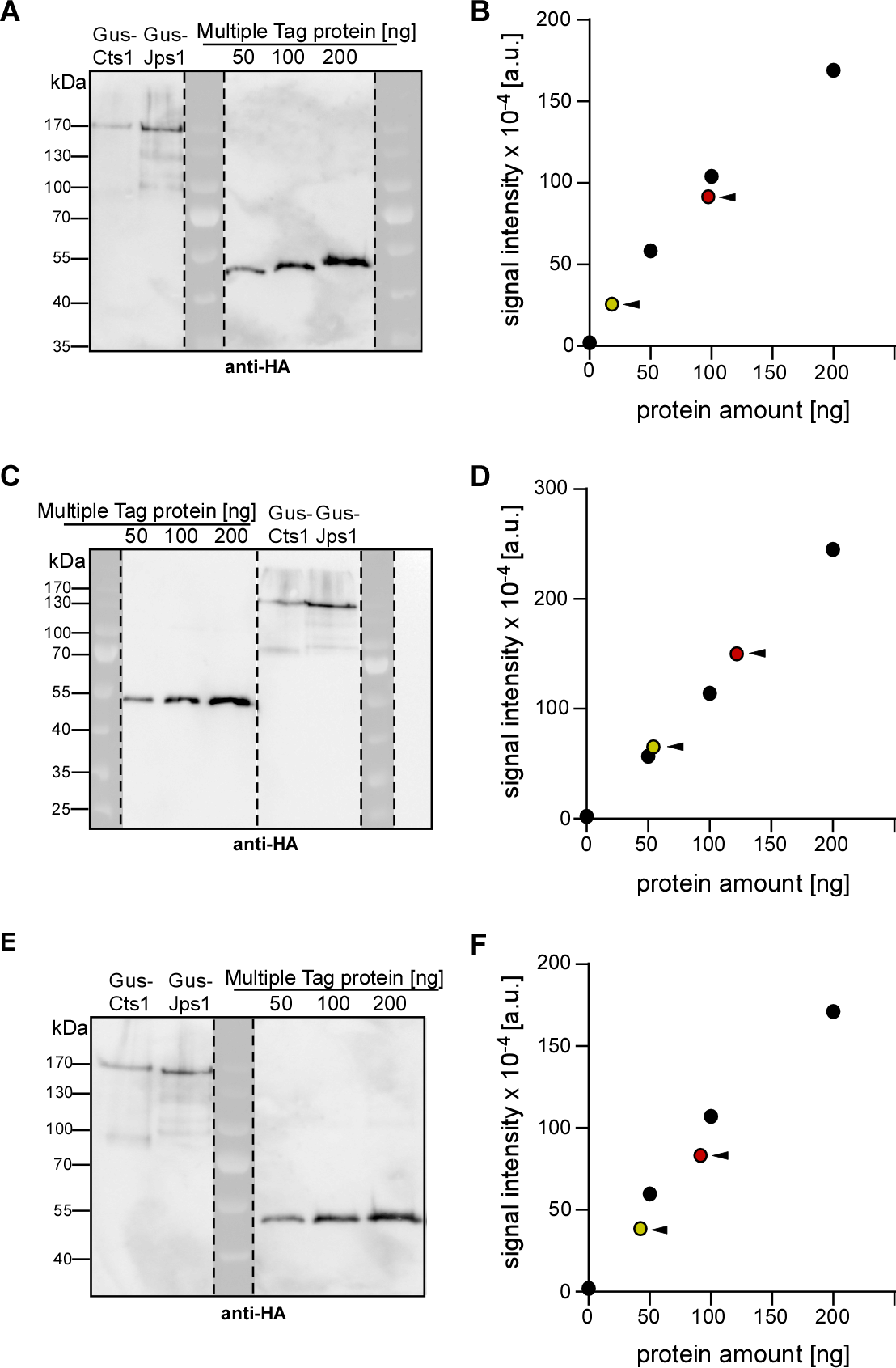


**Supplementary Figure 4. Quantitative Western blot analysis of Gus-Cts1 and Gus-Jps1 secretion. (A, C, E)** Total extracellular protein was concentrated via TCA precipitation from 1 ml of supernatant of Gus-Jps1 and Gus-Cts1 producing strains and subjected to SDS-PAGE and subsequent Western blot analysis using antibodies directed against the HA tag. Standards of Multiple Tag protein (GenScript Biotech, Piscataway, NJ, USA)) were loaded onto the gels equalling amounts of 50, 100, 200 and 500 ng. Expected protein sizes: Gus-Cts1, 134 kDa; Gus-Jps1, 145 kDa. **(B, D, F)** Western blot signals of Multiple Tag protein standards were quantified using Image Studio Lite (LI-COR Biosciences, Lincoln, NE, USA) and used to extrapolate protein amounts of Gus-Jps1 and Gus-Cts1 in the supernatant. Standard curve derived from signal strenghts of the Multiple Tag protein is depicted by black dots, yellow dots indicate signals obtained and cognate protein amounts calculated from linear regression for Gus-Cts1, red dots indicate signal obtained and cognate protein amount calculated from linear regression for Gus-Jps1. Figure pairs **(A/B; C/D; E/F)** each depict Western blot and derived quantification curves of one replicate.
