# Supplementary figures and images for: A Novel Potent Carrier for Unconventional Protein Export in Ustilago maydis
Source: Front Cell Dev Biol. 2022 Jan 10;9:816335. doi: 10.3389/fcell.2021.816335 (PMC8784666; doi:10.3389/fcell.2021.816335)

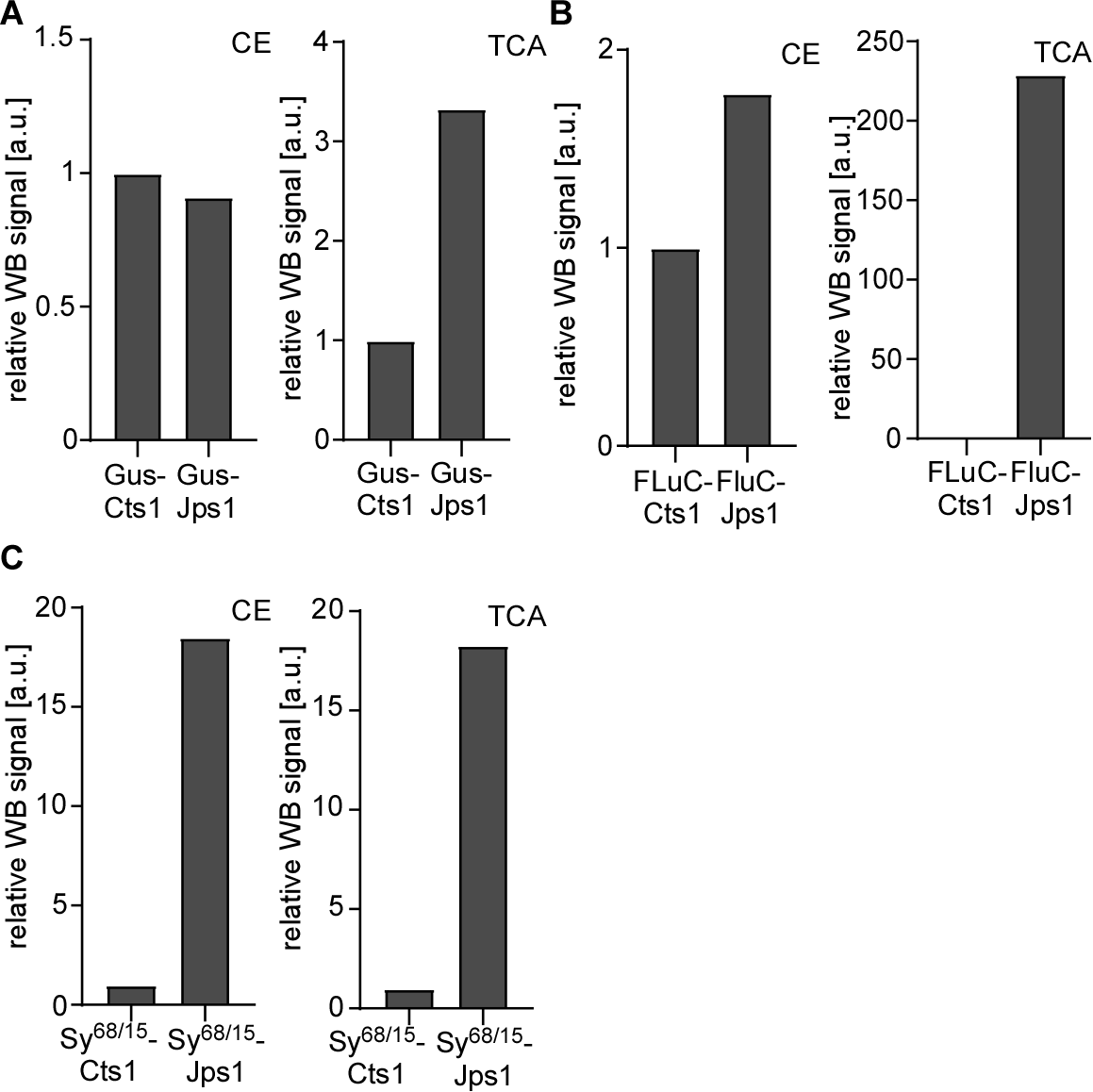

Supplement: Supplementary file 3 [file Image3.TIF]

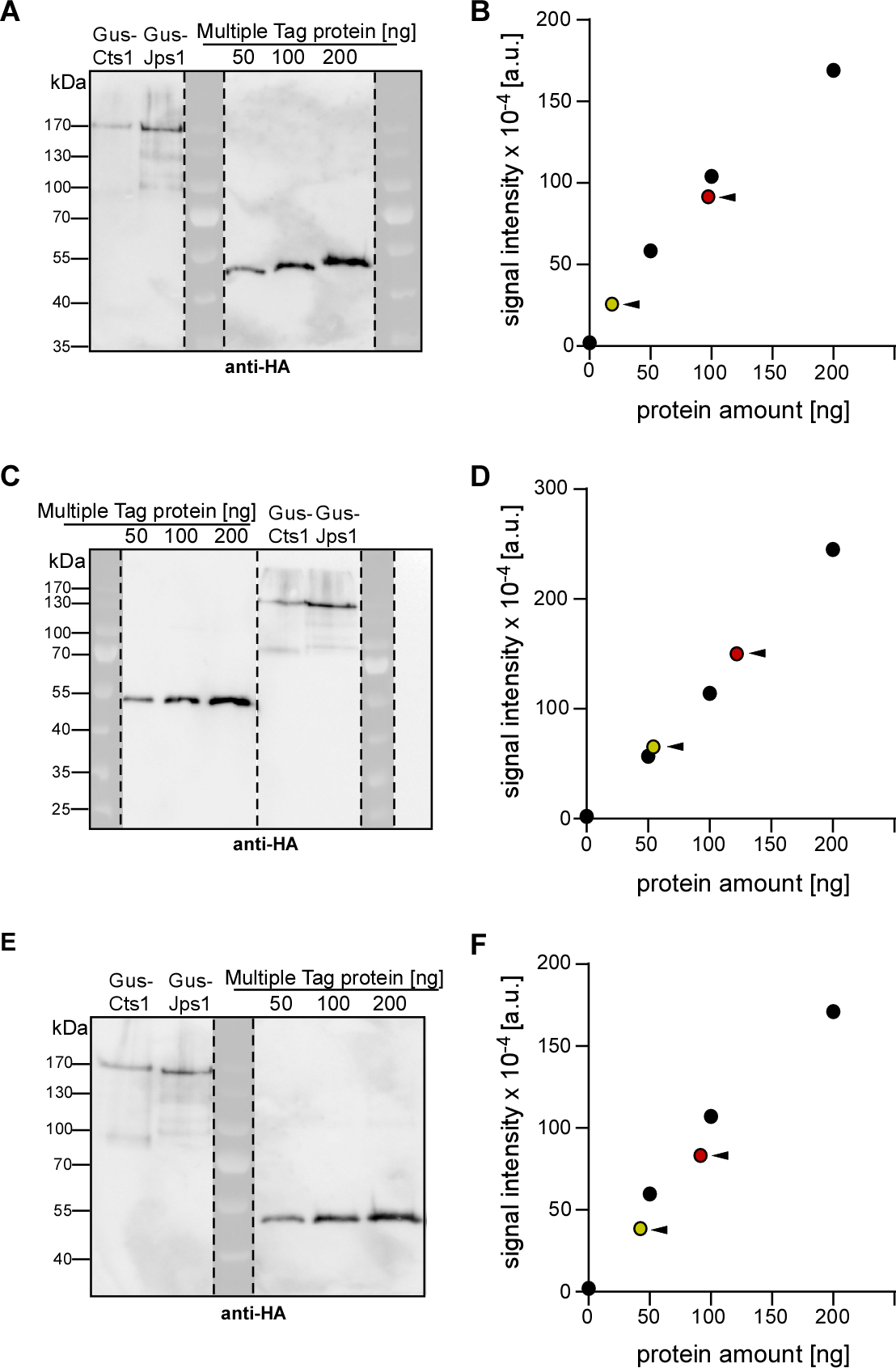

Supplement: Supplementary file 4 [file Image4.TIF]

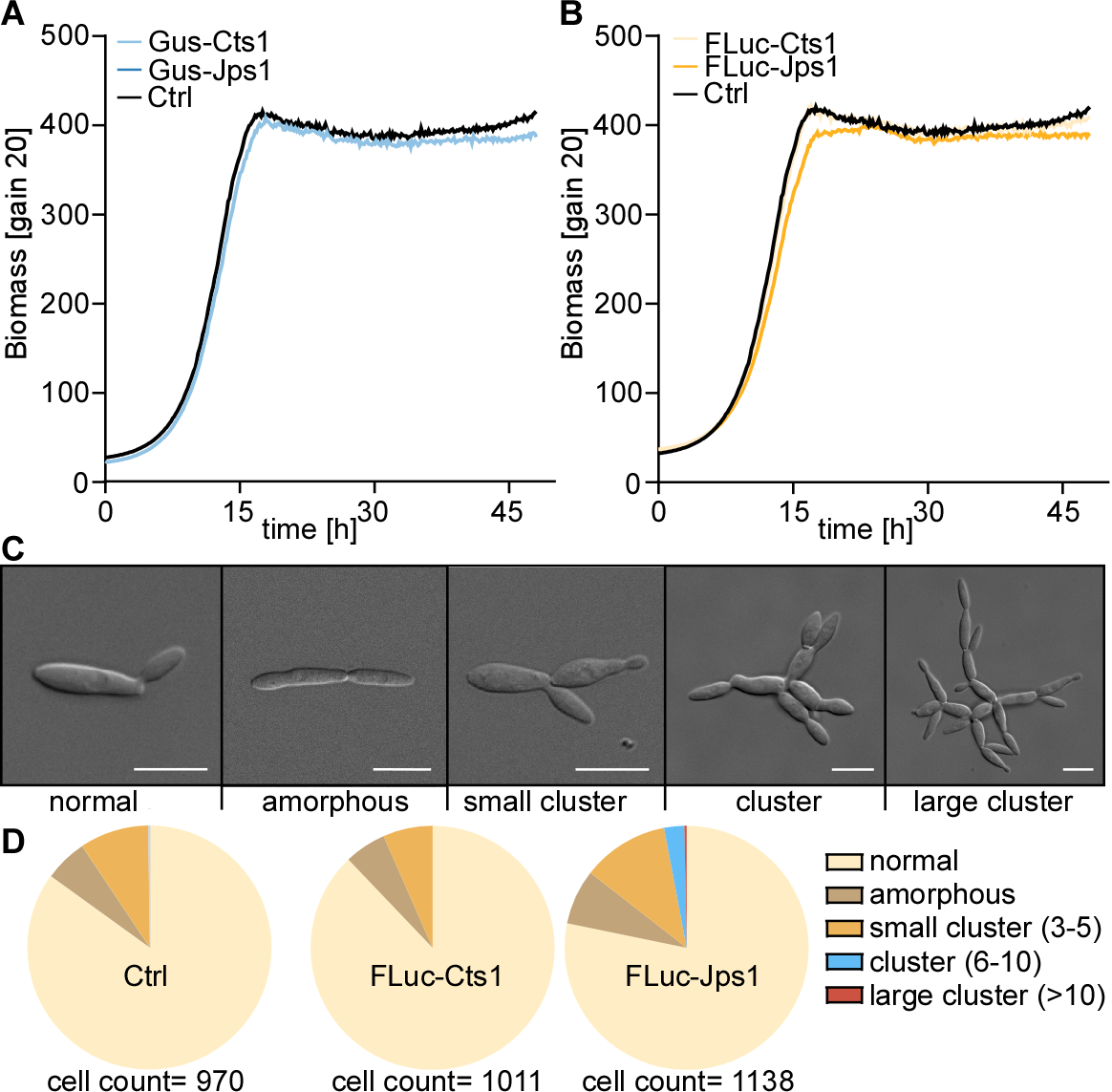

Supplement: Supplementary file 5 [file Image2.TIF]

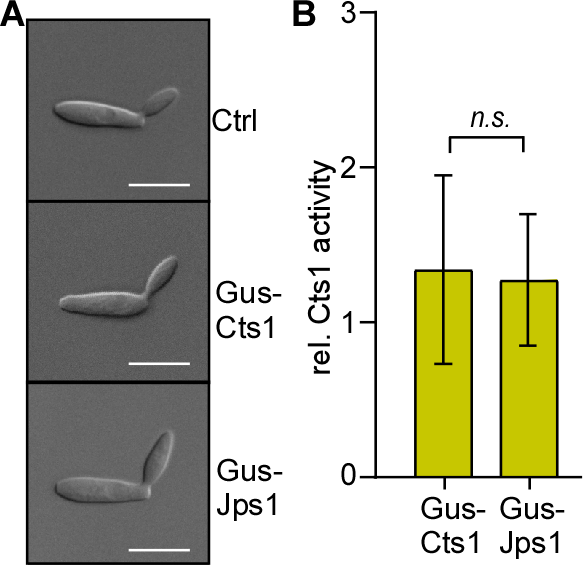

Supplement: Supplementary file 6 [file Image1.TIF]
