# Supplementary material for: A Novel Potent Carrier for Unconventional Protein Export in Ustilago maydis
Source: Front Cell Dev Biol. 2022 Jan 10;9:816335. doi: 10.3389/fcell.2021.816335 (PMC8784666; doi:10.3389/fcell.2021.816335)
Supplement: Supplementary file 9 [file DataSheet1.docx]

# Tables

**Table 1.** DNA oligonucleotides used in this study.

| **Designation** | **Nucleotide sequence (5´- 3´)** |
| --- | --- |
| oMB372_jps1_fw | TTAGGCGCGCCATGCCAGGCATCTCC |
| oMB373_jps1_rev | TTAGGGCCCTTAGGATTCCGCATCGATTGGGG |
| oMF502_ip_fw | ACGACGTTGTAAAACGACGGCCAG |
| oMF503_ip_rev | TTCACACAGGAAACAGCTATGACC |
| oAB297_fluc_fw | AAATTGGATCCATGGAGGACGCCAAGAACATCAAG |
| oAB298_fluc_rev | AATAGGCCGCGTTGGCCACGGCGATCTTGCCACCCTT |
| oAB908_sy^#15^_fw | ATATAGGATCCATGGCGGCCCATCACCACCATCACCACCATCACCACCATCATATGCAGGTGCAGCTCG |
| oAB909_sy^#15^_rev | ATATAACTAGTCGAGACGGTGACCTGGGTGC |
| oCD234_sy^#68^_fw | CTACCTTACTCTATCAGGATCATGCAGGTGCAGCTCGTCG |
| oCD235_sy^#68^_rev | GGTGATGGGCCGCCATGGATCCCGAGACGGTGACCTGGGTGC |

**Table 2.** *U. maydis* strains used in this study.

| **Strains** | **Relevant genotype/ Resistance** | **Strain collection no. (UMa^1^)** | **Plasmids transformed / Resistance^2^** | **Manipulated locus** | **Pro-genitor (UMa^1^)** | **Reference** |
| --- | --- | --- | --- | --- | --- | --- |
| **AB33** | *a2 P_nar_bW2bE1*  PhleoR | 133 | pAB33 | *b* | FB2 (55) | (Brachmann et al. 2001) |
| **AB33 Gus-Cts1** | *a2 P_nar_bW2bE1* PhleoR  *ip^S^[P_oma_gus:shh:cts1]ip^R^*CbxR | 1289 | pUMa2113/ CbxR | *ip* | 133 | [(Sarkari et al. 2014)](file:///C:\Users\Magnus\Desktop\Vorträge,%20Poster%20und%20Paper\Publizierte%20Einheiten\Philipp%20et%20al.%202022%201\test.xlsx#RANGE!_ENREF_33) |
| **AB33don3Δ/Gus-Cts1** | *a2 P_nar_bW2bE1* PhleoR  *ip^S^[P_oma_gus:shh:cts1]ip^R^* CbxR  *umag_don3Δ*_HygR | 1742 | pUMa2717/ HygR | *umag_05543 (don3)* | 1289 | [(Aschenbroich et al. 2019)](file:///C:\Users\Magnus\Desktop\Vorträge,%20Poster%20und%20Paper\Publizierte%20Einheiten\Philipp%20et%20al.%202022%201\test.xlsx#RANGE!_ENREF_1) |
| **AB33don3Δ** | *a2 P_nar_bW2bE1* PhleoR  *umag_don3Δ*_HygR | 2028 | pUMa2717/HygR | *umag_05543 (don3)* | 133 | [(Aschenbroich et al. 2019)](file:///C:\Users\Magnus\Desktop\Vorträge,%20Poster%20und%20Paper\Publizierte%20Einheiten\Philipp%20et%20al.%202022%201\test.xlsx#RANGE!_ENREF_1) |
| **AB33don3Δ/ P_crg_don3-gfp/Gus-Cts1** | *a2 P_nar_bW2bE1* PhleoR  *ip^S^[P_oma_gus:shh:cts1]ip^R^* CbxR  *umag_don3Δ*_HygR  *upp1::[P_crg_don3:gfp]* NatR | 2302 | pUMa3330/ NatR | *umag_02178* (*upp1*) | 1742 | [(Aschenbroich et al. 2019)](file:///C:\Users\Magnus\Desktop\Vorträge,%20Poster%20und%20Paper\Publizierte%20Einheiten\Philipp%20et%20al.%202022%201\test.xlsx#RANGE!_ENREF_1) |
| **AB33P8∆Gus-Cts1** | *a2 P_nar_bW2bE1* PhleoR  *FRT10[um04641Δ::hyg]*  *FRT11[um03947Δ]*  *FRT6[um03975Δ]*  *FRT5[um04400Δ]*  *FRT3[um11908Δ]*  *FRT2[um00064Δ]*  *FRTwt[um02178Δ]*  *FRT1[um04926Δ]* HygR  *ip^S^[P_oma_gus:shh:cts1]ip^R^* CbxR | 2418 | pUMa2113 | *Ip* | 2413 | [(Terfrüchte et al. 2018)](file:///C:\Users\Magnus\Desktop\Vorträge,%20Poster%20und%20Paper\Publizierte%20Einheiten\Philipp%20et%20al.%202022%201\test.xlsx#RANGE!_ENREF_40) |
| **AB33don3Δ/ Gus-Jps1** | *a2 P_nar_bW2bE1* PhleoR  *ip^S^[P_oma_gus:shh:cts1]ip^R^* CbxR  *umag_don3Δ*_HygR | 2734 | pUMa3012 | *Ip* | 2028 | This study |
| **AB33don3Δ/ P_crg_don3-gfp/Gus-Jps1** | *a2 P_nar_bW2bE1* PhleoR  *ip^S^[P_oma_gus:shh:cts1]ip^R^* CbxR  *umag_don3Δ*_HygR  *upp1::[P_crg_don3:gfp]* NatR | 2776 | pUMa3330/ NatR | *umag_02178* (*upp1*) | 2734 | This study |
| **AB33P8∆Gus-Jps1** | *a2 P_nar_bW2bE1* PhleoR  *FRT10[um04641Δ::hyg]*  *FRT11[um03947Δ]*  *FRT6[um03975Δ]*  *FRT5[um04400Δ]*  *FRT3[um11908Δ]*  *FRT2[um00064Δ]*  *FRTwt[um02178Δ]*  *FRT1[um04926Δ]* HygR  *ip^S^[P_oma_gus:shh:jps1]ip^R^* CbxR | 2900 | pUMa3012 | *Ip* | 2413 | this study |
| **AB33P8∆ FLuc-Cts1** | *a2 P_nar_bW2bE1* PhleoR  *FRT10[um04641Δ::hyg]*  *FRT11[um03947Δ]*  *FRT6[um03975Δ]*  *FRT5[um04400Δ]*  *FRT3[um11908Δ]*  *FRT2[um00064Δ]*  *FRTwt[um02178Δ]*  *FRT1[um04926Δ]* HygR  *ip^S^[P_oma_fluc:shh:cts1]ip^R^* CbxR | 3151 | pUMa4131 | *Ip* | 2413 | this study |
| **AB33P8∆ FLuc-Jps1** | *a2 P_nar_bW2bE1* PhleoR  *FRT10[um04641Δ::hyg]*  *FRT11[um03947Δ]*  *FRT6[um03975Δ]*  *FRT5[um04400Δ]*  *FRT3[um11908Δ]*  *FRT2[um00064Δ]*  *FRTwt[um02178Δ]*  *FRT1[um04926Δ]* HygR  *ip^S^[P_oma_fluc:shh:jps1]ip^R^* CbxR | 3214 | pUMa4566 | *ip* |  | this study |
| **AB33P8∆Sy#68/#15-Cts1** | *a2 P_nar_bW2bE1* PhleoR  *FRT10[um04641Δ::hyg]*  *FRT11[um03947Δ]*  *FRT6[um03975Δ]*  *FRT5[um04400Δ]*  *FRT3[um11908Δ]*  *FRT2[um00064Δ]*  *FRTwt[um02178Δ]*  *FRT1[um04926Δ]* HygR  *ip^S^[P_oma_antirbdsybody#68:his:antirbdsybody#15:ha:cts1]ip^R^* CbxR | Ux1 | pUx1 | *ip* | 2413 | this study |
| **AB33P8∆Sy#68/#15-Jps1** | *a2 P_nar_bW2bE1* PhleoR  *FRT10[um04641Δ::hyg]*  *FRT11[um03947Δ]*  *FRT6[um03975Δ]*  *FRT5[um04400Δ]*  *FRT3[um11908Δ]*  *FRT2[um00064Δ]*  *FRTwt[um02178Δ]*  *FRT1[um04926Δ]* HygR  *ip^S^[P_oma_antirbdsybody#68:his:antirbdsybody#15:ha:jps1]ip^R^* CbxR | Ux8 | pUx8 | *ip* | 2413 | this study |

^1^ Internal strain collection numbers (UMa/Ux codes)

^2^ Plasmids generated in our working group are integrated in a plasmid collection and termed pUMa or pUx plus a number as 4-digit number as identifier.
